# Gastrointestinal Digestion Impact on Phenolics and Bioactivity of Tannat Grape Pomace Biscuits

**DOI:** 10.3390/molecules30153247

**Published:** 2025-08-02

**Authors:** Victoria Olt, Jessica Báez, Romina Curbelo, Eduardo Boido, Eduardo Dellacassa, Alejandra Medrano, Adriana Maite Fernández-Fernández

**Affiliations:** 1 Laboratorio de Bioactividad y Nanotecnología de Alimentos, Departamento de Ciencia y Tecnología de Alimentos, Facultad de Química, Universidad de la República, General Flores 2124, Montevideo 11800, Uruguay; volt@fq.edu.uy (V.O.); jbaez@fq.edu.uy (J.B.); 2 Graduate Program in Chemistry, Facultad de Química, Universidad de la República, General Flores 2124, Montevideo 11800, Uruguay; 3 Área Analítica Orgánica, Departamento de Química Orgánica, Facultad de Química, Universidad de la República, General Flores 2124, Montevideo 11800, Uruguay; rominacurbelo12@gmail.com (R.C.); edellac@fq.edu.uy (E.D.); 4 Área Enología y Biotecnología de la Fermentación, Departamento de Ciencia y Tecnología de Alimentos, Facultad de Química, Universidad de la República, General Flores 2124, Montevideo 11800, Uruguay; eboido@fq.edu.uy

**Keywords:** bioaccessibility, bioactivity, biscuits, in vitro digestion, phenolic compounds, tannat grape pomace

## Abstract

The search for natural sources of bioactive compounds with health-promoting properties has intensified in recent years. Among these, Tannat grape pomace (TGP), a primary byproduct of winemaking, stands out for its high phenolic content, although its bioactivity may be affected during gastrointestinal digestion. This study aimed to evaluate the impact of in vitro digestion on the antioxidant (ABTS, ORAC-FL, intracellular ROS inhibition), anti-diabetic (α-glucosidase inhibition), anti-obesity (lipase inhibition), and anti-inflammatory (NO inhibition) properties of five sugar-free biscuits formulated with varying percentages of TGP and sucralose. No significant differences were observed in the bioaccessible fractions (BFs, representing the compounds potentially released in the small intestine) between control biscuits and those enriched with TGP, suggesting limited release of phenolics at this stage. Conversely, the colonic fractions (CFs, simulating the material reaching the colon) from biscuits with higher TGP content exhibited greater bioactivities. HPLC-DAD-MS analysis of the CF from the biscuit containing 20% TGP and 4% sucralose revealed a high content of procyanidin trimers, indicating the persistence of these specific phenolic compounds after in vitro digestion. These findings suggest that TGP-enriched biscuits may deliver health benefits at the colonic level and support their potential application in the formulation of functional foods. Further microbiota and in vivo studies should be assessed to confirm the latter.

## 1. Introduction

The increasing consumer demand for natural and sustainable ingredients has significantly intensified the scientific interest in food byproducts as promising sources of bioactive compounds with potential health benefits [1]. As a result, emerging research areas are focusing on studying the health effects of winery byproducts. Particular attention is given to their role in improving several disorders related to oxidative stress and inflammation, which are risk factors for the development of chronic diseases [2]. Grape pomace is the main solid byproduct of winemaking. It is composed primarily of seeds and peels and is a rich and natural source of health-promoting compounds, including dietary fiber and phenolic compounds that remain in the pomace after juice extraction [3,4]. Among grape varieties, Tannat is the most emblematic red wine grape cultivated in Uruguay [5], and its pomace (TGP) has been identified as a rich source of dietary fiber (64%) and phenolic compounds. The phenolic profile of TGP mainly includes anthocyanins, flavonols, and flavan-3-ols, which are widely recognized for their biological activity [6]. Anthocyanins are the pigments responsible for the intense purple-red color of grapes. In TGP the most abundant is malvidin-3-O-(6′-p-coumaroyl) glucoside which is known for its strong bioactive properties [6,7]. Flavonols are also present in significant amounts, with quercetin-3-O-glucoside being particularly abundant in TGP. These compounds and their metabolites have shown protective effects against oxidative stress, contributing to the prevention of chronic conditions such as cardiovascular and metabolic disorders [6,8]. Flavan-3-ols, which contribute to the organoleptic properties of grapes and wine, are mainly represented by procyanidin trimers in TGP and are associated with cardiometabolic benefits [6,9]. The abundance of these bioactive compounds in TGP supports its potential as a multifunctional ingredient for the development of health-oriented food products [10].

Despite TGP having such bioactive properties, the beneficial effects on health depend on the extent to which these compounds are released during gastrointestinal digestion (bioaccessibility) and subsequently absorbed into systemic circulation (bioavailability) [11,12]. It is estimated that only 5–10% of dietary polyphenols are absorbed in the small intestine, primarily those in aglycone forms [13]. Most polyphenols reach the colon, where they interact with the gut microbiota or are excreted [11]. Moreover, when phenolic compounds are incorporated into food products, they can interact with macromolecules present in the food matrix, which may affect their bioaccessibility, bioavailability, and consequently the impact they could have on health [6,14]. For all these reasons, investigating the interaction between polyphenols and the intestinal microenvironment is essential for clarifying the mechanisms underlying the health benefits of polyphenol-rich foods [12,15].

Considering that Tannat is the most emblematic grape variety in our country [5] and that its pomace has shown great potential as a functional ingredient [6], we hypothesized that biscuits enriched with TGP should present phenolic compounds and bioactive properties after in vitro digestion. This would support their potential as a functional food. The present study aimed to evaluate the functional properties of sugar-free biscuits enriched with different concentrations of TGP and sucralose, following in vitro gastrointestinal digestion simulation. The research focused on evaluating the antioxidant, anti-diabetic, anti-obesity, and anti-inflammatory properties of the fractions generated from in vitro digestion. These include the bioaccessible fraction, representing the portion potentially available for absorption in the small intestine, and the colonic fraction, corresponding to the material that reaches the colon for potential microbial metabolism. Furthermore, phenolic compounds present in the colonic fraction of the most bioactive formulation were identified using HPLC-DAD-MS to explore potential associations between chemical composition and biological activity.

## 2. Results and Discussion

### 2.1. Phenolic Compounds Profile in the Colonic Fraction (CF)

**Figure 1:**
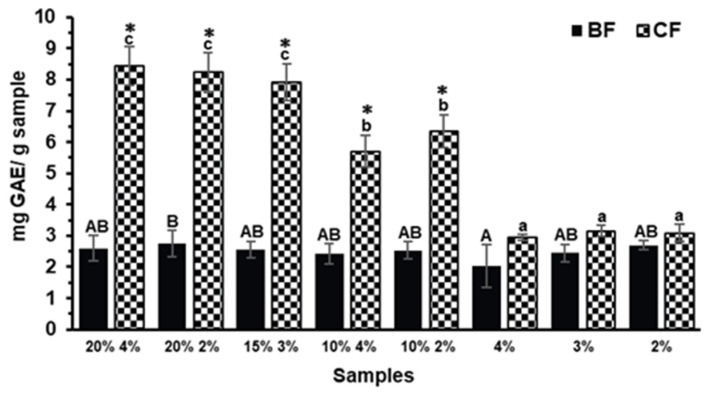
Total phenol content (TPC) of the bioaccessible and colonic fractions (BFs and CFs, respectively) of the different Tannat grape pomace (TGP) biscuits (TGP% sucralose%: 20% 4%, 20% 2%, 15% 3%, 10% 4%, and 10% 2%) and their control biscuits (sucralose%: 4%, 3%, and 2%). For BFs bars denote the mean values minus the digestion control (digestion without any sample), and for CFs bars denote the mean values. Error bars denote the standard deviation. Different lowercase letters represent significant differences between the CFs of the different biscuit formulations (Tukey test, *p* < 0.05). Different capital letters represent significant differences between the BFs of the different biscuit formulations (Tukey test, *p* < 0.05). Asterisks (*) indicate significant differences between BF and CF within the same biscuit formulation (*t*-test, *p* < 0.05).

**Figure 2:**
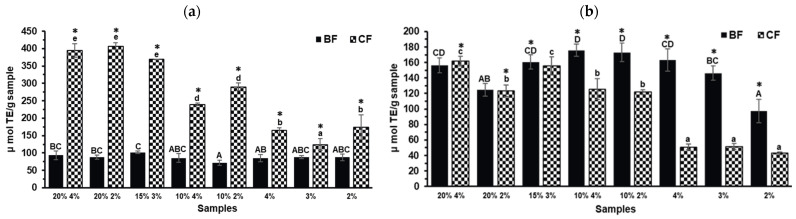
Antioxidant capacity measured by (**a**) ABTS and (**b**) ORAC-FL of the bioaccessible and colonic fractions (BFs and CFs, respectively) of the different TGP biscuits (TGP% sucralose%: 20% 4%, 20% 2%, 15% 3%, 10% 4%, and 10% 2%) and their control biscuits (sucralose%: 4%, 3%, and 2%). For BFs bars denote the mean values minus the digestion control (digestion without any sample), and for CFs bars denote the mean values. Error bars denote the standard deviation. Different lowercase letters represent significant differences between the CFs of the different biscuit formulations according to the Tukey test (*p* < 0.05). Different capital letters represent significant differences between the BFs of the different biscuit formulations according to the Tukey test (*p* < 0.05). Asterisks (*) indicate significant differences between BF and CF within the same biscuit formulation (*t*-test, *p* < 0.05).

Phenolic compounds identified in the CF of TGP and the biscuit containing 20% TGP and 4% sucralose (20% 4%) are presented in Table 1. HPLC-DAD-MS chromatograms (Figure S1) and identification of the peaks (Table S1) are shown in the Supplementary Material. No phenolic compounds were detected in the CF of the control biscuit with 4% sucralose. The 20% 4% biscuit formulation was selected for analysis due to its high TPC and antioxidant activity as measured by ABTS and ORAC-FL (Figure 1 and Figure 2).

In the CF of the 20% 4% biscuit, the predominant compound was procyanidin trimer (Rt 9.10 min), followed by malvidin-3-O-(6′-p-coumaroyl)glucoside, representing procyanidin trimers 45% of the total area. In contrast, the CF of TGP showed malvidin-3-O-glucoside, malvidin-3-O-(6′-p-coumaroyl)glucoside, procyanidin trimer (Rt 9.0 min), and (+)-catechin as the main compounds, in descending order. Except for the procyanidin trimer (Rt 9.10 min), all identified compounds had higher peak areas in the CF of TGP than in the biscuit CF. This compound was tentatively identified as a procyanidin trimer based on its retention time, UV-Vis absorption spectrum, and characteristic mass fragmentation pattern obtained by HPLC-DAD-MS. Previous studies have suggested that polymeric procyanidins can interact with digestive enzymes, which may lead to their precipitation and retention in the insoluble fraction during simulated digestion [16]. A decrease in flavan-3-ols, specifically polymeric procyanidins, has been previously reported in the BF of grape pomace extracts [17]. This supports the idea that these compounds are not efficiently released into the soluble phase and instead remain in the CF. In addition to precipitation, the polymerization of phenolic compounds during digestion due to pH changes is proposed as another possible hypothesis to explain their higher relative content [11]. This predominant presence of procyanidin trimers obtained in the present study could be beneficial for consumer health. Once in the colon, procyanidins and their monomeric units may undergo microbiota metabolism, altering their bioavailability and resulting in the production of low molecular weight phenolic acids with potential health benefits [18,19]. Regarding other grape byproducts, some phenolic compounds (syringic and cinnamic acids, ε-viniferin, myricetin, and naringenin) from grapevine bunch stem and cane from the Malbec grape cultivar have been found to be highly bioaccessible [20]. However, procyanidins (monomers, dimers, and trimers) bioaccessibility was very low or completely missed. Accordingly, a cereal-based food matrix with extracts from other grape byproducts has shown proantocyanidins and total flavonoids with low bioaccessibility [21]. Similarly, the bioaccessibility studies of heat-treated skim goat-milk powder fortified with grape-pomace-seed extract showed low recovery of flavan-3-ols and phenolic acids [22].

A comparative analysis between the biscuit before [6] and after in vitro digestion (Table 1) revealed a 56% increase in the total peak area in the CF of the digested biscuit compared to the undigested sample. This increase in the total peak area may be partially explained by the release of phenolic compounds during digestion, likely due to the disruption of interactions with macromolecules in the biscuit matrix [23]. These interactions may hinder full compound recovery during extraction from undigested samples, resulting in an underestimation of phenolic content prior to digestion [17,24]. Regarding TGP, several phenolic compounds were originally present [cis-caftaric acid, protocatechuic acid, trans-caftaric acid, p-coumaroyl hexose, two procyanidin trimers (Rt 16.02 and 16.32), procyanidin dimers (B2, B7, and galloylated forms), and flavonol glycosides (myricetin-3-O-galactoside, and quercetin-3-O-galactoside, syringetin-3-O-glucoside)] [6] were not detected in the CF of TGP after digestion. Moreover, these compounds were also not detected in the undigested biscuit [6]. This suggests a possible degradation or transformation of these compounds during thermal processing and/or gastrointestinal digestion, where enzymes and pH changes may alter their stability and bioavailability [25,26].

Conversely, some compounds—such as (–)-epicatechin gallate, delphinidin-3-O-glucoside, and cyanidin-3-O-glucoside—were not originally detected in TGP but were identified in its CF, suggesting that they may have been released from the food matrix or formed as transformation products during digestion [17,24].

To our knowledge, this is the first study to identify phenolic compounds in the insoluble fraction of digested biscuits formulated with grape pomace, highlighting the potential of winemaking byproducts as sources of bioactive compounds.

### 2.2. Total Phenolic Content (TPC)

The evaluation of total phenolic content (TPC) across the BFs and CFs aimed to determine the extent to which phenolic compounds were released or retained following in vitro digestion. As shown in Figure 1, the TPC in BFs of biscuits with and without TGP addition showed no significant differences (*p* > 0.05), indicating that phenolic compounds were not effectively released during the small intestinal phase of digestion. In contrast, the TPC of the CFs differed significantly (*p* < 0.05) among formulations, with biscuits containing 20% and 15% TGP exhibiting the highest TPC values. The presence of non-bioaccessible phenolic compounds reaching the colon along with indigestible polysaccharides has been previously reported [27,28]. Although, to our knowledge, there is no available data regarding TPC in the CF of grape pomace, similar behavior has been observed in other agro-industrial byproducts such as avocado peel. It has been reported that a large proportion of phenolic compounds (approximately 66%), including condensed and hydrolysable tannins, resist gastrointestinal digestion and remain in the non-digestible fraction, allowing them to reach the colon and undergo microbial metabolism [29].

To evaluate the effect of digestion, the TPC of the biscuit containing 20% TGP and 4% sucralose (20% 4%) was compared before [6] and after simulated gastrointestinal digestion (Figure 1). In our previous work, the undigested biscuit showed a TPC of 1.85 ± 0.04 mg GAE/g [6]. After digestion, the BF exhibited a non-significant increase to 2.60 ± 0.42 mg GAE/g (*p* > 0.05), whereas the CF reached a significantly higher value of 8.44 ± 0.62 mg GAE/g (*p* < 0.05), indicating a higher concentration of phenolic compounds retained in the CF. The lack of significant change in the BF suggests limited release of phenolic compounds during the upper gastrointestinal phase. This result is consistent with previous findings showing that most dietary phenolics have low absorption rates in the small intestine due to their strong interaction with food matrix components [13,30]. The increase in the CF may reflect their partial release or concentration in the insoluble fraction as a result of digestion.

### 2.3. Antioxidant Capacity

Phenolic compounds can be transformed in the digestive tract [31], making it crucial to determine the mechanism of action of antioxidant compounds once they transit through the gastrointestinal tract. Regarding the antioxidant compounds present in the CF (Figure 2), the same trend as the TPC results was observed. Biscuit formulations containing a higher percentage of TGP (20% and 15%) showed the highest ABTS values (Figure 2a). On the other hand, the compounds in the BF of the biscuits with TGP predominantly exhibited the HAT mechanism of action by the ORAC-FL method (Figure 2b), reaching similar values to the ones of CF. The antioxidant capacity in the BF cannot be attributed solely to the presence of phenolic compounds derived from TGP as a similar antioxidant capacity was observed for the BF of the biscuits with and without TGP (control biscuits) (*p* > 0.05). During digestion, proteins are hydrolyzed, releasing bioactive peptides [32], which have been shown to possess antioxidant, anti-diabetic, anti-hypertensive, and anti-inflammatory properties [33]. The findings of this study indicate that the antioxidant compounds in the BF after in vitro digestion may include bioactive peptides derived from the proteins in the flour and eggs of the biscuits [32].

To the best of our knowledge, there are no previous reports on the antioxidant capacity of the BF and CF after in vitro digestion of products with grape pomace. However, similar analyses have been conducted on other grape byproducts as well as other polyphenolic sources. Consistent with the findings of this study, the BF of a yogurt with Tannat grape skin showed similar values to the control yogurt (without the byproduct) [34]. However, in the same study, the BF of a biscuit with Tannat grape skin showed increased ABTS values when compared to the control biscuit (without the byproduct) [34]. Moreover, cereal-based food matrices enriched with extracts from other grape byproducts have been shown to increase the total polyphenol content and to contribute to their antioxidant capacity after in vitro digestion [21]. In contrast to the findings of the present study, results reported by Odriozola-Serrano et al. [35] showed that after in vitro gastrointestinal digestion of a rosehip extract, the CF exhibited lower antioxidant capacity (measured by DPPH and FRAP) compared to its BF. Conversely, after chemical-enzymatic digestion of avocado peel, no antioxidant capacity was observed in its BF, as measured by DPPH, ABTS, and FRAP. However, antioxidant capacity was observed in the non-digestible fraction, suggesting that phenolic compounds may reach the colon during gastrointestinal digestion [29]. Considering these reports from other plant sources, it is important to highlight the role of the matrix in the release process of phenolic compounds during digestion, making bioaccessibility studies necessary for each case. In addition, the phenolic composition must be taken into consideration as well due to different stability and chemical transformations during digestion [30].

**Figure 3:**
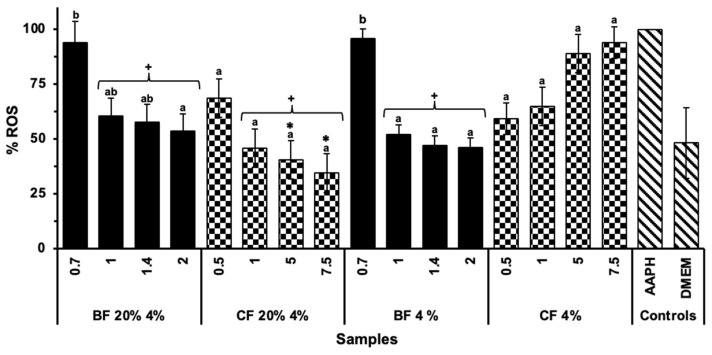
AAPH (1 mM)-induced ROS formation in normal human colon cells (CCD 841 CoN) treated with different concentrations of the bioaccessible and colonic fractions (BFs and CFs, respectively) of 20% 4% biscuit (TGP% sucralose%) and its control biscuit (4% sucralose) without TGP. DMEM was used as the negative control and AAPH as the positive control. A digestion without any sample was also assessed as a control. Bars represent the mean values, and error bars represent the standard error. Different letters denote significant differences between concentrations (mg/mL) of the same sample (Tukey, *p* < 0.05). Asterisks (*) indicate significant differences between the same concentrations (mg/mL) of both BF and CF samples (*t*-test, *p* < 0.05). + Indicates significant differences between the sample and the positive control (AAPH) (*t*-test, *p* < 0.05).

In addition to determining the mechanisms of action of antioxidant compounds, it is crucial to evaluate their impact in cell lines, as these methods may not accurately reflect the potential effects of antioxidants in living systems [36]. In this context, the BFs of both biscuits (same concentration of BF 20% 4%, and 4%) showed no significant differences (*p* > 0.05) between them in their capacity to reduce intracellular ROS formation (Figure 3). However, both BF significantly reduced (*p* < 0.05) intracellular ROS formation, reaching basal levels (negative control). This effect was observed when compared to the positive control (AAPH) and the digestion control (digestion without any sample), indicating that the observed effect may be exerted by the compounds from the ingredients of the biscuit generated during digestion. The BFs may contain bioactive peptides released from the proteins in the biscuit ingredients by the in vitro digestion [32], which could interact with cells and trigger antioxidant responses by influencing cellular signaling pathways that regulate the expression of antioxidant enzymes [37]. Conversely, the CF of the 20% 4% biscuit significantly (*p* < 0.05) reduced intracellular ROS formation when compared to the CF of the control biscuit and the positive control (AAPH). This suggests that the compounds present in the CF of the TGP biscuit may play a crucial role in counteracting the effects of oxidative stress in the colon cells. Similar protective effects against intracellular ROS generation were observed in previous studies, where the bioaccessible fraction of yogurt with Tannat grape skin reduced ROS formation in normal human colon fibroblasts (CCD-18Co cells). This effect could be partially attributed to the phenolic compounds and bioactive peptides released from the yogurt matrix during digestion [34], emphasizing the influence of the food matrix on the bioaccessibility of phenolic compounds. According to Martins et al. [38], polyphenols present in a grape pomace extract obtained from mixed varieties (Syrah and Seibel) can effectively eliminate peroxyl radicals in the extracellular compartment under AAPH-induced oxidation before these can react with the probe in the intracellular compartment. Thus, this could be a possible explanation of the mechanism of action of TGP polyphenols in the reduction of intracellular ROS formation in colon cells. To the best of our knowledge, this is the first study to investigate the antioxidant effect of the insoluble fraction after in vitro digestion on normal human colon cells.

### 2.4. Anti-Diabetic and Anti-Obesity Capacities

The evaluation of the anti-diabetic and anti-obesity properties of the biscuits after in vitro digestion aimed to elucidate the interactions between the compounds present in the digestive fractions (BF and CF) and key gastrointestinal tract enzymes, such as α-glucosidase and pancreatic lipase.

**Figure 4:**
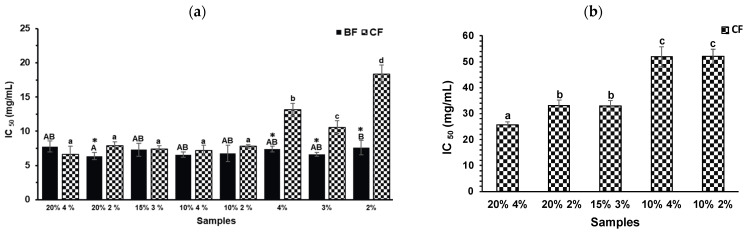
(**a**) α-glucosidase inhibition of the bioaccessible and colonic fractions (BFs and CFs, respectively) of the different TGP biscuits (TGP% sucralose%: 20% 4%, 20% 2%, 15% 3%, 10% 4%, and 10% 2%) and their control biscuits (sucralose%: 4%, 3%, and 2%). (**b**) Pancreatic lipase inhibition of the colonic fractions (CFs) of the different formulations of TGP biscuits. The BFs of TGP biscuits, as well as both BFs and CFs of control biscuits, are not shown due to the absence of measurable inhibitory activity under the assay conditions. Bars denote the mean values, and error bars the standard deviation. Different lowercase letters represent significant differences between the CFs of the different biscuit formulations according to the Tukey test (*p* < 0.05). Different capital letters represent significant differences between the BFs of the different biscuit formulations according to the Tukey test (*p* < 0.05). Asterisks (*) indicate significant differences between the CF and BF of the same biscuit formulation (*t*-test, *p* < 0.05).

Regarding α-glucosidase inhibition (Figure 4a), no significant differences were observed in the BF across all formulations, including the controls (*p* > 0.05). This suggests that the inhibitory activity in the BF is not primarily driven by polyphenols from TGP but may instead be attributed to compounds released from the biscuit matrix during digestion. For instance, peptides derived from egg white hydrolysates have been reported to inhibit α-glucosidase [39]. In contrast, CFs from TGP biscuits exhibited significantly higher inhibition (*p* < 0.05) than their respective controls, suggesting a contribution from phenolic compounds retained in the colonic fraction. The inhibitory activity of polyphenols is closely related to their chemical structure, which facilitates interaction with enzyme active sites or substrates [40]. Notably, the procyanidin trimer, the predominant compound identified in the CF of the 20% TGP biscuit (Table 1), may play a central role. Proanthocyanidins, due to their polymeric nature and multiple interaction sites, have demonstrated potent inhibitory effects on α-amylase and α-glucosidase [41].

For pancreatic lipase inhibition (Figure 4b), neither the BFs of any formulation nor the CFs of control biscuits exhibited measurable inhibitory activity. However, the CFs of formulations containing 20% and 15% TGP showed significant inhibition, indicating that phenolic compounds in the colonic fraction may be responsible. Polyphenols, particularly proanthocyanidins from grape peel and catechins from tea, have been shown to inhibit fat-digesting enzymes, including pancreatic lipase [42]. Therefore, the compounds identified in the CF by HPLC-DAD-MS (especially procyanidins) may underlie the observed anti-obesity potential.

### 2.5. Anti-Inflammatory Capacity

**Figure 5:**
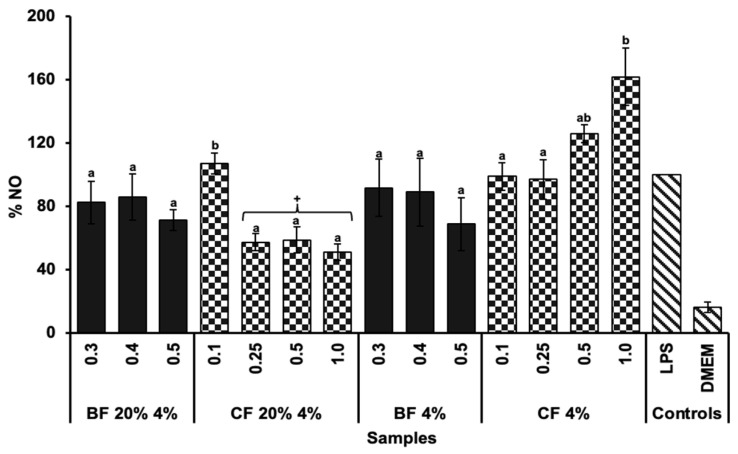
LPS (1 µg/mL)-induced NO production in RAW 264.7 cells treated with the bioaccessible and colonic fractions (BF and CF, respectively) of a biscuit with 20% TGP and 4% sucralose (20% 4%) and its control biscuit (4%) without TGP. DMEM was used as the negative control and AAPH as the positive control. A digestion without any sample was also assessed as a control. Bars represent the mean values, and error bars represent the standard error. Different letters denote significant differences between concentrations (mg/mL) of the same sample (Tukey, *p* < 0.05). + Indicates significant differences between the sample and the positive control (LPS) (*T*-test, *p* < 0.05).

The anti-inflammatory activity of the biscuit samples was evaluated by measuring the inhibition of nitric oxide (NO) production in LPS-stimulated RAW 264.7 macrophages (Figure 5). No significant differences (*p* > 0.05) were observed between the bioaccessible fractions (BFs) of biscuits with and without TGP in terms of NO inhibition. Furthermore, neither BF demonstrated a significant reduction (*p* > 0.05) in NO levels compared to the positive control (LPS). In contrast, the CF of the 20% 4% formulation showed significant differences (*p* < 0.05) by reducing NO production compared to the CF of its control biscuit (4%) and the positive control (LPS). This result suggests that the phenolic compounds provided by the TGP may play a role in modulating NO production [43,44]. Previous studies have shown that phenolic compounds possess anti-inflammatory capacity by reducing NO production under inflammatory conditions [44]. Specifically, in line with the present results, it has been reported that a hydroalcoholic-acid extract of Tannat grape pomace peel reduces NO production in RAW 264.7 macrophages exposed to LPS stimulation [45], as well as the bioaccessible compounds of this extract [46]. A possible explanation may be that certain compounds, such as polyphenols, could have the capacity to modify the expression or functionality of nitric oxide synthase (NOS), which is the enzyme involved in NO production [47]. Among the various phenolic compounds, the procyanidin trimer could be responsible for intervening in this metabolic pathway, as it has been reported for its ability to influence the expression of different nitric oxide synthase isoforms [48,49]. In addition, anthocyanins, another prominent group of phenolic compounds in TGP, have been reported to have anti-inflammatory activity. Studies suggest that anthocyanins can modulate inflammatory responses by inhibiting the production of NO through downregulation of NOS expression [50]. Therefore, the presence of anthocyanins in the CF of the biscuits could further explain the observed reduction in NO production, as their synergistic action with other phenolic compounds may contribute to the anti-inflammatory effects seen in this study [51]. Further studies are needed to confirm this hypothesis.

## 3. Materials and Methods

### 3.1. Materials and Reagents

Analytical grade solvents and reagents were employed for the physicochemical analyses, in vitro digestion, and cell experiments. The buffer salts, dimethyl sulfoxide (DMSO), Folin reagent, 2,2′-azinobis-(3-ethylbenzothiazoline-6-sulfonic acid) diammonium salt (ABTS), 6-hydroxy-2,5,7,8-tetramethylchroman-2-carboxylic acid (Trolox), fluorescein (FL) disodium salt, 2,2′-azobis(2-methylpropionamidine) dihydrochloride (AAPH), α-glucosidase, 4-methylumbelliferyl-α-D-glucopyranoside (4-MUG), pancreatic lipase, 4-methylumbelliferyl oleate (4-MUO), human α-amylase (1500 U/mL), porcine pepsin (25,000 U/mL), porcine pancreatin (800 U/mL), bovine bile, 3-(4,5-dimethylthiazol-2-yl)-2,5-diphenyltetrazolium bromide (MTT), 2′,7′-dichlorofluorescein diacetate (DCFH-DA), and lipopolysaccharide from E. coli O55 (LPS) were sourced from Sigma-Aldrich (St. Louis, MO, USA).

Normal human colon cells (CCD 841 CoN) and mouse macrophage cells (RAW 264.7) were procured from the American Type Culture Collection (ATCC, Manassas, VA, USA). CCD 841 CoN cells were cultured in Dulbecco’s Modified Eagle Medium (DMEM) supplemented with 20% (*v*/*v*) heat-inactivated fetal bovine serum (FBS) and 1% (*v*/*v*) antibiotics (penicillin-streptomycin, 1:1), all supplied by Gibco Laboratory (Invitrogen Co., Grand Island, NY, USA), whereas RAW 264.7 cells were supplemented with 10% (*v*/*v*) heat-inactivated fetal bovine serum (FBS).

### 3.2. Samples

Five biscuit formulations were developed using a two-factorial design with central points, following the procedure described by Olt et al. [6]. The formulations varied in their content of TGP and sucralose, as follows: 20% TGP with 4% sucralose, 20% TGP with 2% sucralose, 15% TGP with 3% sucralose, 10% TGP with 4% sucralose, and 10% TGP with 2% sucralose. All percentages were expressed on a total wet dough basis.

TGP was obtained fresh from the Bouza wine cellar (Montevideo, Uruguay) immediately after the winemaking process. The pomace was dried in a conventional oven at 50 °C for 24 h, ground using a household electric coffee grinder, and stored at −20 °C in a freezer until use [45].

Biscuits were formulated as previously reported by Olt et al. [6], using wheat flour, sunflower oil, baking powder, eggs, sucralose, salt, and TGP. All ingredients were sourced from local suppliers in Montevideo, Uruguay. The dough from each formulation was rolled out to a uniform thickness of approximately 5 mm and cut into circular discs with a diameter of 4 cm. Biscuits were baked in a conventional oven at 180 °C for 12 min, cooled to room temperature, ground using a household grinder, and stored at −20 °C until further analysis.

### 3.3. Simulated Digestion of the Biscuits

The in vitro gastrointestinal digestion simulation of the biscuits was carried out using the INFOGEST protocol [52]. The five biscuit formulations (TGP% sucralose%: 20% 4%, 20% 2%, 15% 3%, 10% 4%, and 10% 2%) and their control biscuits (without TGP) (sucralose%: 4%, 3%, and 2%) were digested in triplicate. Firstly, biscuits were subjected to the oral phase, being diluted with simulated salivary and human α-amylase at pH 7. Samples were incubated with shaking for 2 min in a water bath at 37 °C. For the gastric phase, simulated gastric fluid with porcine pepsin and lipase was added to the oral sample at pH 3. Samples were incubated with shaking for 2 h in a water bath at 37 °C. For the small intestinal phase, the gastric samples were added with the simulated intestinal fluid, pancreatin, and bile salts at pH 7. The samples were also incubated with shaking for 2 h in a water bath at 37 °C. Finally, enzymatic inactivation was carried out, followed by centrifugation (9000 rpm, 10 min, 4 °C). Two fractions were obtained per biscuit: the soluble fraction, referred to as the bioaccessible fraction (BF), consisting of components potentially absorbable; and the insoluble fraction, referred to as the colonic fraction (CF), comprising unabsorbed compounds that may reach the colon. The BFs were used directly after the in vitro gastrointestinal digestion simulation, whereas the CFs were lyophilized and stored at −20 °C until further analysis. A digestion assay without biscuits was conducted as a control.

### 3.4. Identification of Phenolic Compounds in the Colonic Fraction

Phenolic compound extraction was performed following a modified protocol based on Peña-Vázquez et al. [53]. Briefly, 50 mg of the colonic fraction (CF) from the biscuit containing 20% TGP and 4% sucralose (20% 4%), its corresponding control biscuit (4% sucralose), and the TGP powder were mixed with 1 mL of a methanol:acidified water solution (80:20, *v→v*). Samples were subjected to sonication for 90 min, vortexed for 1 min, and then centrifuged at 9500 rpm for 10 min at 4 °C. The supernatant was collected, and the residue was re-extracted using 0.5 mL of the same solvent under identical conditions. The supernatants from both steps were combined and stored at −20 °C for subsequent HPLC-DAD-MS analysis, following the methodology described by Olt et al. [6].

HPLC-DAD-MS analyses were performed on a Kinetex C18-EVO reverse-phase column (Phenomenex) maintained at 35 °C. The mobile phase consisted of 0.1% trifluoroacetic acid in water (A) and acetonitrile (B) with the following gradient elution program: 0–100% A for 3 min; 4–30% B over 50 min; 30–98% B over 5 min; followed by 2 min of isocratic elution at 98% B. The flow rate was set at 1.3 mL/min. Detection was carried out at 280 nm. The HPLC system was coupled to a Shimadzu Triple Quadrupole mass spectrometer equipped with an electrospray ionization (ESI) source (Shimadzu, Tokyo, Japan). This approach was adopted to enable relative comparisons among samples rather than absolute quantification.

### 3.5. Total Phenolic Content

Total phenolic content (TPC) in the gastrointestinal simulated fractions—BFs and CFs—was determined using the Folin–Ciocalteu method [45]. Absorbance was measured at 750 nm after 30 min of incubation in the dark, using a microplate reader (Multiskan FC, Thermo Scientific, Waltham, MA, USA). Gallic acid was used as the calibration standard, and results were expressed as milligrams of gallic acid equivalents (mg GAE) per gram of sample.

For TPC analysis, BFs were used directly after the in vitro digestion process. CFs, on the other hand, were resuspended in a DMSO:distilled water solution (1:1, *v*/*v*) to enhance the extraction of phenolic compounds. This extraction procedure was selected based on preliminary tests with different DMSO:water proportions.

### 3.6. Antioxidant Capacity

The antioxidant capacity was assessed using the ABTS assay, which evaluates single electron transfer (SET) capacity, and the ORAC-FL assay, which evaluates hydrogen atom transfer (HAT) capacity [45]. For the ABTS assay, absorbance was recorded at 750 nm after 10 min of incubation in the dark using a microplate reader (Multiskan FC, Thermo Scientific, Waltham, MA, USA). For the ORAC-FL assay, fluorescence (λ excitation = 485 nm, λ emission = 520 nm) was recorded every minute for 80 min at 37 °C using a Varioskan™ Lux microplate reader (SkanIt RE 5.0 software, Thermo Scientific, Waltham, MA, USA). In both assays, Trolox was used as the calibration standard, and results were expressed as µmol of Trolox equivalents (TE) per gram of sample. For ABTS, the Trolox calibration curve was constructed [Absorbance vs. µmol/mL TE], followed by interpolation of the values of Absorbance to the curve and division of the sample concentration in g/mL. For ORAC-FL, the Trolox calibration curve was constructed [area under the curve (AUC) of trolox vs. µmol/mL TE], followed by interpolation of the values of AUC to the curve and division of the sample concentration in g/mL. BFs were used as obtained after the in vitro digestion, while CFs were extracted as described in Section 3.5.

To evaluate intracellular antioxidant activity, the inhibition of reactive oxygen species (ROS) production under induced oxidative stress conditions was assessed in CCD 841 CoN cells (normal human colon epithelial cells), following the protocol by Fernández-Fernández et al. [34] with minor modifications. BFs (100 mg/mL) were used directly, while CFs were prepared in ethanol (95%):water (EtOH:H_2_O, 80:20 *v*/*v*; final EtOH concentration 8% in the well). Sample solutions were filtered and diluted in DMEM-P/S without fetal bovine serum (FBS). Sample concentrations were selected based on the cytotoxicity (MTT assay) performed previous to the evaluation of intracellular antioxidant activity, considering 80% as the minimum cell viability. Cells were seeded at 1 × 10^4^ cells/well (100 µL/well) in a sterile 96-well translucent microplate with a lid and incubated at 37 °C for 24 h. Afterwards, cells were pre-treated with 100 µL of different concentrations of filtered samples (BFs and CFs) at 37 °C for 24 h. Cells were incubated with DCFH-DA probe (5 mg/mL in DMSO) for 30 min after supernatant removal. Once the 30 min incubation ended and cells were washed with PBS, 100 µL of filtered samples with the oxidative agent AAPH (1 mM) were added to each well and incubated at 37 °C for 2 h. Intracellular ROS production was quantified by measuring fluorescence (λ excitation = 485 nm, λ emission = 525 nm) using a Varioskan™ Lux microplate reader (SkanIt RE 5.0 software, Thermo Scientific, Waltham, MA, USA). Data normalization was performed using the MTT assay [46]. Positive control presented 1 mM AAPH in DMEM-P/S without FBS, which represented 100% ROS formation. Negative control presented DMEM-P/S without FBS. All determinations were performed in triplicate and in 3 different cell passages.

### 3.7. Anti-Diabetic and Anti-Obesity Capacity

The anti-diabetic and anti-obesity capacities of the samples were evaluated through enzymatic inhibition assays targeting α-glucosidase and pancreatic lipase, respectively. In both assays, fluorescence (λ excitation = 360 nm, λ emission = 460 nm) was measured using a Varioskan™ Lux microplate reader (SkanIt RE 5.0 software, Thermo Scientific, Waltham, MA, USA) after 30 min of incubation at 37 °C. α-Glucosidase inhibition was assessed using 4-methylumbelliferyl-α-D-glucopyranoside (4-MUG) as the substrate, while pancreatic lipase inhibition was evaluated using 4-methylumbelliferyl oleate (4-MUO). Reference inhibitors (acarbose and orlistat for α-glucosidase and pancreatic lipase, respectively) were used as reaction controls. The degree of inhibition was expressed as the concentration of sample required to inhibit 50% of the enzyme activity (IC_50_, mg/mL) [54]. Inhibition percentages were calculated using the following equation:

(1) $$\%\;Inhibition=\frac{Fluorescence\;with\;no\;inhibitor-\;Fluorescence\;sample}{Fluorescence\;with\;no\;inhibitor}\times \;100$$

where the fluorescence without an inhibitor was composed of the enzyme and the substrate, indicating the maximum of enzyme activity, and the fluorescence sample was composed of enzyme, substrate, and sample.

BFs were used as obtained after in vitro digestion, while CFs were extracted as described in Section 3.5.

### 3.8. Anti-Inflammatory Capacity

To evaluate the anti-inflammatory capacity of samples, the nitric oxide (NO) production in RAW 264.7 cells (macrophages from mouse tumor) was determinate as an indicator of chronic inflammation [45]. Sample concentrations were selected based on the cytotoxicity (MTT assay) performed previous to the evaluation of anti-inflammatory capacity, considering 80% as the minimum cell viability. RAW 264.7 cells were seeded at 8 × 10^4^ cells/well (100 µL/well) in a sterile 96-well translucent microplate with a lid and incubated at 37 °C for 24 h (until confluence). Cells were pre-treated with different concentrations of filtered samples (100 µL/well), prepared as described for the intracellular ROS formation assay (Section 3.6). After 24 h, supernatant was removed, and cells were treated for 24 h with 150 µL of filtered samples with LPS (1 µg/mL) as an inducer of inflammation. After incubation, 100 µL of cell supernatants were transferred to another 96-well plate and mixed with 100 µL of Griess reagent, which reacts with organic nitrites. After 15 min incubation in the dark at room temperature, absorbance at 550 nm was measured in a VarioskanTM Lux (SkanIt RE 5.0 software, Thermo Scientific, Waltham, MA, USA). To normalize data by the viable cells’ number, an MTT assay was performed by adding 20 μL/well of MTT (6 mM) reagent and incubating for 30 min [46]. Positive control presented 1 µg/mL LPS in DMEM-P/S without FBS, which represented 100% of NO production. Negative control presented DMEM-P/S without FBS. All determinations were performed in triplicate and in three different cell passages.

### 3.9. Statistical Analysis

Three independent experiments were performed for each assay. Results were expressed as mean ± standard deviation. Statistical differences between the mean values of the studied parameters of the different samples were determined by the Tukey test (*p* < 0.05). In cell studies, the statistical differences between mean values of samples were determined by the *t*-test (*p* < 0.05). The program used for statistical analyses was Infostat v. 2020.

## 4. Conclusions

The results from this research provide valuable insights into the impact of in vitro gastrointestinal digestion on the release and retention of phenolic compounds from biscuits formulated with varying percentages of TGP. The BFs showed similar levels of bioactivity among themselves and compared to their respective control biscuits. In contrast, the CFs of biscuits with higher TGP content (20% and 15%) exhibited significantly greater antioxidant and enzyme inhibitory activities. These findings indicate that phenolic compounds may be selectively retained in the CF during digestion.

In particular, the CF of the biscuit containing 20% TGP and 4% sucralose showed significantly higher inhibition of lipase and α-glucosidase compared to the rest of the biscuits. Furthermore, the CF of the biscuit with 20% TGP and 4% sucralose showed a significant reduction of intracellular ROS and NO formation compared to the CF of the control biscuit, as well as compared to the BFs, suggesting the potential of phenolic compounds to counteract oxidative and nitrosative stress. HPLC-DAD-MS analysis revealed the high areas of procyanidin trimers in the CF, which may contribute to its observed bioactivities.

This study expands current knowledge on the bioaccessibility and bioactivity of biscuits enriched with TGP, combining in vitro gastrointestinal digestion, cell assays, and phenolic compound identification. It underscores the critical role of matrix–compound interactions in modulating phenolic bioaccessibility and highlights the potential of TGP to contribute bioactive compounds to the colonic environment. Further studies (in vitro and in vivo) are needed to identify the phenolic compounds in the BFs, as well as to investigate the metabolism of CFs in the colon and the phenolic compound transformation by intestinal microbiota.

## Figures and Tables

**Table 1:** Data on identification of phenolic compounds in the colonic fraction (CF) of TGP and of the biscuit with 20% of TGP and 4% of sucralose (20% 4%) analyzed by HPLC-DAD-MS. Rt: Retention time.

	Compound		CF TGP		CF Biscuit 20% 4%	
			Rt (min)	Area	Rt (min)	Area
**Chromatogram 280 nm**	**Phenolic acids**	Trans-caftaric acid	6.911	16,981		
	**Flavan-3-ols**	Procyanidin trimer C2	4.62	4624	4.867	1495
		Procyanidin dimer B1	7.334	9819	7.224	2166
		Procyanidin dimer B3	7.795	63,891	7.768	21,267
		(+)-Catechin	8.335	130,356	8.274	41,044
		Procyanidin trimer	9.008	185,897	9.109	271,845
		Procyanidin trimer	10.554	99,312	10.509	30,335
		Procyanidin dimer B4	11.397	35,471	11.299	9612
		Procyanidin dimer B6	11.952	22,016	11.826	3210
		(−)-Epicatechin	12.442	84,897	12.379	28,992
		Galloylated procyanidin dimer	15.364	19,076	15.346	14,467
		(−)-Epicatechin gallate	18.389	6723		
	**Flavonols**	Myricetin-3-O-glucoside	21.816	9007		
		Quercetin-3-O-glucoside	23.388	60,556	23.385	4589
		Quercetin-7-O-neohesperidoside	28.161	26,998	28.204	8073
		Quercetin (aglycone)	35.086	44,860	35.165	15,043
**Chromatogram 520 nm**	**Anthocyanins**	Delphinidin-3-O-glucoside	14.221	6325	14.236	1490
		Cyanidin-3-O-glucoside	16.208	2038		
		Petunidin-3-O-glucoside	17.65	54,033	17.709	6960
		Peonidin-3-O-glucoside	19.669	36,138	19.771	1733
		Malvidin-3-O-glucoside	20.771	398,827	20.925	42,507
		Petunidin-3-O-(6′-acetyl)glucoside	26.555	10,056	26.61	1621
		Peonidin-3-O-(6′-acetyl)glucoside	28.907	6793		
		Malvidin-3-O-(6′-acetyl)glucoside	29.508	39,730	29.555	4603
		Delfinidin-3-O-(6′-p-coumaroyl)glucoside	30.471	26,936	30.527	9299
		Malvidin-3-O-(6′-caffeoyl)glucoside	32.356	30,092	32.39	3240
		Cyanidin-3-O-(6′-p-coumaroyl)glucoside	32.766	8846	32.812	2113
		Petunidin-3-O-(6′-p-coumaroyl)glucoside	33.448	55,245	33.495	17,043
		Peonidin-3-O-(6′-p-coumaroyl)glucoside	35.865	37,621	35.913	10,862
		Malvidin-3-O-(6′-p-coumaroyl)glucoside	36.19	362,321	36.236	121,228

## Data Availability

The datasets generated and analyzed during this study are available from the authors on request.

## Supplementary Materials

The following supporting information can be downloaded at: https://www.mdpi.com/article/10.3390/molecules30153247/s1, Figure S1: HPLC-DAD chromatograms: (a) CF of TGP; (b) CF of TGP biscuit. The identification of the peaks is listed in Table S1; Table S1: Identification of the peaks in Figure S1.

## Abbreviations

The following abbreviations are used in this manuscript:

4-MUG	4-methylumbelliferyl-α-D-glucopyranoside
4-MUO	4-methylumbelliferyl oleate
AAPH	2,2′-Azo- bis (2-methylpropionamidine) dihydrochloride
ABTS	2,2′-Azino-bis (3-ethylbenzothiazoline-6-sulphonic acid)
CCD 841 CoN	Cell line isolated from colon tissue of a healthy donor
FBS	Fetal bovine serum
GAE	Gallic acid equivalents
DCFH-DA	2′,7′-dichlorodihydrofluorescein diacetate
DMEM	Dulbecco’s modified eagle medium
DMSO	Dimetilsulfoxide
HAT	Hydrogen atom transfer
HPLC-DAD-MS	High-performance liquid chromatography with diode array detection and mass spectrometry
IC_50_	Inhibitory concentration 50%
LPS	Lipopolysaccharide
MTT	3-(4,5-dimethylthiazole-y)-2,5-diphenyltetrazolium bromide
NO	Nitric oxide
ORAC	Oxygen radical absorbance capacity
RAW 264.7	Mouse macrophage cell line
ROS	Reactive oxygen species
TPC	Total phenolic content
TGP	Tannat grape pomace
TROLOX	6-hydroxy-2,5,7,8-tetramethylch-roman-2-acid
SET	Single electron transfer
