# Supplementary material for: Gastrointestinal Digestion Impact on Phenolics and Bioactivity of Tannat Grape Pomace Biscuits
Source: Molecules. 2025 Aug 2;30(15):3247. doi: 10.3390/molecules30153247 (PMC12348079; doi:10.3390/molecules30153247)
Supplement: Supplementary file 1 [file molecules-30-03247-s001.zip › molecules-3695964-supplementary.pdf]

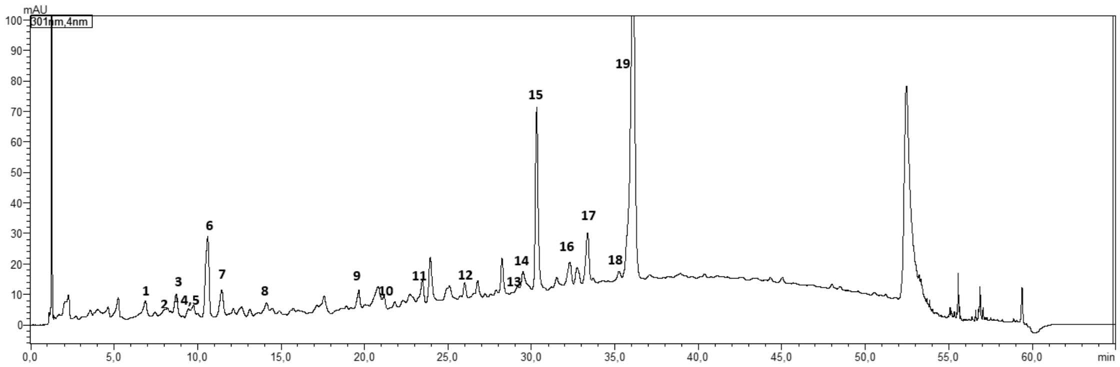

(**a**)

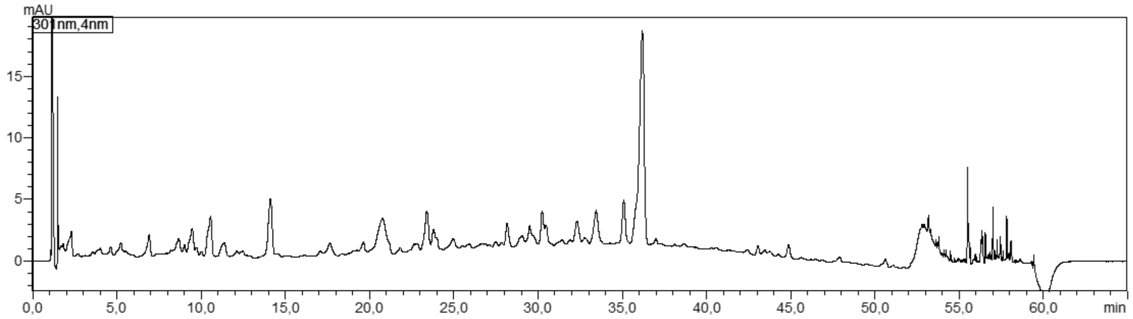

(**b**)

Figure S1. HPLC-DAD chromatograms: (**a**) CF of TGP; (**b**) CF of TGP biscuit. The identification of the peaks is listed in Table S1.

Table S1. Identification of the peaks in Figure S1.

Peak number	compound	[M + H]$^+$	λmax
**1**	*trans*-Caftaric acid	311	250, **329**
**2**	Procyanidin dimer B1	579	**278**
**3**	(+) Catechin	291	233**, 278**
**4**	Procyanidin dimer B4	579	**278**
**5**	Procyanidin dimer B6	579	**278**
**6**	Procyanidin trimer	867	**278**
**7**	(-)-Epicatechin	291	234, **278**
**8**	Petunidin-3-*O*-glucoside	479	278, 344, 373, **527**
**9**	Malvidin-3-O-glucoside	493	277, 348, **527**
**10**	Peonidin-3-*O*-glucoside	463	276, 315, 362, **516**
**11**	Quercetin-3-*O*-glucoside	465	256, **354**
**12**	Petunidin-3-O-(6′-acetyl)glucoside	521	280, **522**
**13**	Malvidin-3-O-(6′-acetyl)glucoside	535	278, 350, **530**
**14**	Peonidin-3-O-(6′-p-coumaroyl)glucoside (*trans*)	609	283, 313, **526**
**15**	Delphinidin-3-O-(6′-p-coumaroyl)glucoside	611	282, 313, **531**
**16**	Malvidin-3-O-(6′-caffeoyl)glucoside (*trans*)	655	282, 313, **532**
**17**	Petunidin-3-O-(6′-p-coumaroyl)glucoside (trans)	625	282, 313, **532**
**18**	Quercetin aglycone	303	255, **370**
**19**	Malvidin-3-O-(6′-p-coumaroyl)glucoside (*cis*)	639	280, 301, **535**
